# The challenge of bridging the gap between researchers and policy makers: experiences of a Health Policy Research Group in engaging policy makers to support evidence informed policy making in Nigeria

**DOI:** 10.1186/s12992-016-0209-1

**Published:** 2016-11-04

**Authors:** Benjamin Uzochukwu, Obinna Onwujekwe, Chinyere Mbachu, Chinenye Okwuosa, Enyi Etiaba, Monica E. Nyström, Lucy Gilson

**Affiliations:** 1 Department of Community Medicine, College of Medicine, University of Nigeria, Enugu-campus, Nigeria; 2 Department of Pharmacology and Therapeutics, College of Medicine, University of Nigeria, Enugu-campus, Nigeria; 3 Health Policy Research Group, College of Medicine, University of Nigeria, Enugu-campus, Nigeria; 4 Department of Health Administration and Management, College of Medicine, University of Nigeria, Enugu-campus, Nigeria; 5 Department of Learning, Informatics, Management and Ethics (LIME), Karolinska Institutet, 171 77 Stockholm, Sweden; 6 Department of Public Health and Clinical Medicine, Umeå university, 901 87 Umeå, Sweden; 7 Health Policy and Systems Division, School of Public Health and Family Medicine, University of Cape Town, Cape Town, South Africa

**Keywords:** Getting research into policy and practice, Nigeria

## Abstract

**Background:**

Getting research into policy and practice (GRIPP) is a process of going from research evidence to decisions and action. To integrate research findings into the policy making process and to communicate research findings to policymakers is a key challenge world-wide. This paper reports the experiences of a research group in a Nigerian university when seeking to ‘do’ GRIPP, and the important features and challenges of this process within the African context.

**Methods:**

In-depth interviews were conducted with nine purposively selected policy makers in various organizations and six researchers from the universities and research institute in a Nigerian who had been involved in 15 selected joint studies/projects with Health Policy Research Group (HPRG). The interviews explored their understanding and experience of the methods and processes used by the HPRG to generate research questions and research results; their involvement in the process and whether the methods were perceived as effective in relation to influencing policy and practice and factors that influenced the uptake of research results.

**Results:**

The results are represented in a model with the four GRIPP strategies found: i) stakeholders’ request for evidence to support the use of certain strategies or to scale up health interventions; ii) policymakers and stakeholders seeking evidence from researchers; iii) involving stakeholders in designing research objectives and throughout the research process; and iv) facilitating policy maker-researcher engagement in finding best ways of using research findings to influence policy and practice and to actively disseminate research findings to relevant stakeholders and policymakers.

The challenges to research utilization in health policy found were to address the capacity of policy makers to demand and to uptake research, the communication gap between researchers, donors and policymakers, the management of the political process of GRIPP, the lack of willingness of some policy makers to use research, the limited research funding and the resistance to change.

**Conclusions:**

Country based Health Policy and Systems Research groups can influence domestic policy makers if appropriate strategies are employed. The model presented gives some direction to potential strategies for getting research into policy and practice in the health care sector in Nigeria and elsewhere.

## Background

The importance of getting research evidence into policy and practice (GRIPP) is widely acknowledged in literature [1–5]. Over the recent years there has been a proliferation of literature focusing on knowledge utilization and how health policy and practice can be better informed by evidence [6–8]. Two broad issues are involved in the GRIPP process namely; engaging the stakeholders and using evidence in decisions [1]. The goal of GRIPP is to ensure knowledge translation, knowledge transfer, knowledge exchange, research utilization, implementation, diffusion, and dissemination [9]. Other studies have noted that when the public is adequately engaged, there may be increased research uptake especially when civil society organizations, mass media and consumers are involved [10–15]. Adequately engaging the public is only one conclusion of the literature and experience on this topic and not the only solution to the problem.

Several factors have been identified to influence getting research into policy. In addition to linkages between researchers and practitioners, others include attributes of the research (in terms of cost, flexibility, complexity, reversibility, relative advantage, risk, reversibility and comparability), the characteristics of the researcher, practitioner characteristics and the method of dissemination of research results [16].

There are an increasing number of ways to enhance the use of research in health [17]. However, the greatest challenge influencing the use of research in policy making is that research is only one of the inputs to be considered by policy makers amongst many other legitimate inputs [18]. This includes the interest of the actors or decision makers in the policy process which influences the direction of the policy and this poses threats to decision makers’ use of research evidence in taking decision. Other authors have also highlighted best practices in promoting the use of research evidence in policy [1, 19]. Key factors include the timeliness and relevance of findings; the production of credible and trustworthy reports; close personal contacts with policy makers and summaries of findings that present key actionable recommendations.

Despite these best practices, the gap between research and policy and practice is still very wide, especially in low and middle-income countries (LMIC). The failure of take-up of high-quality research evidence by decision makers has been called the gap between research and policy. Researchers have devoted much time and energy to talking about bridging the gap between research and decision making, yet significant gaps still exist between the two. Four misunderstandings between the evidence production and the policy-making effort have been identified. The first point is that researchers and policy makers consider each other’s activity as generating products instead of engaging in processes; second, scientific research attempts to focus the question so that a clear and crisp answer can be provided whereas policy making take other variables such as interests, ideology, values, or opinions into account. Third, decision makers are not sensitive to the incentives that drive researchers like attracting grant money and publishing in peer-reviewed journals and not responding to a current issue before the government policy makers. Fourth, researchers rarely take into account the different audiences that would be audiences for their research [20].

Other researchers have also stated that one of the reasons for the gap is because policy makers rarely convey clear messages about the policy challenges they face in their specific context to allow for timely and appropriate research agendas and researchers on the other hand often produce scientific evidence that is not always tailor-made for application in different contexts [21]. Other common obstacles in this regard are centralized decision making and a policy making culture that gives little importance to evidence based [22].

In Nigeria, the use of research findings by policy makers and communities has been described as very limited and challenging and can be explained by the lack of communication between researchers and policy makers, and the lack of involvement of policy makers and the community in determining the research to be done [23]. Also, the research-to-policy linkages have been generally described as weak [24]. Some factors have been cited for the low uptake of research by Nigerian policymakers including the lack of high-quality research, generally weak and unreliable research institutions and think tanks and apparent disconnect between researchers and policymakers [25]. There is little interaction between policymakers and researchers, thus meaningful discussion of available research findings, their suitability to policy-related problems, and identification of other policy areas requiring research attention is severely lacking [25].

There is also dearth of studies or development in Nigeria that promote evidence-informed policy making involving meetings between researchers and policy makers. Although an innovative effort has been made to bridge the gap between researchers and policy makers [26], such effort concentrated on organizing a 1-day evidence-to-policy forum/workshop and the effect of this intervention on their practice and actually getting research into practice is yet to be evaluated.

Research organizations serve a useful function of linking policy makers, yet the .work of these organizations is sometimes not fully appreciated by policymakers either because researchers do not fully understand the policy process or do not know how to communicate effectively their research findings to policymakers. Therefore, how can country Health Policy and System Research (HPSR) organisations and groups seek to engage policy makers over time and across projects and experiences, and sometimes in collaboration with others? The wider literature focussed on projects because so much of the thinking is linked to donor-funded individual projects. This paper therefore reports the experiences of HPSR group in a Nigerian university when seeking to ‘do’ GRIPP, and describe the important features and challenges of this process within the African context. The research will contribute to the body of knowledge on how to bridge the gap between researchers and policy makers in Nigeria and elsewhere.

### Background of the Health Policy Research Group (HPRG)

The HPRG is a multi-disciplinary group based in the College of Medicine of the University of Nigeria, Enugu-campus. It was founded in 2004 and is dedicated to conducting public health, policy-relevant research and analysis to inform policies, providing policy advice and technical assistance in policy formulation and evaluation and conducting policy dialogues. The HPRG has established regular and wide-ranging communication and information with policy makers in Nigeria and is involved in various capacity building programmes for local policy makers, CSOs and members of the academia. The HPRG has over the years increased the levels of accredited research outputs and publications and is currently a member of several international health policy and system consortia including the Consortium for Health Policy & Systems Analysis in Africa (CHEPSAA) http://www.hpsa-africa.org. and Responsive and Resilient Health Systems (RESYST) http://resyst.lshtm.ac.uk. The HPRG is involved in: Conducting policy-relevant research and analysis; Providing policy advice and technical assistance in policy formulation and evaluation; conducting policy dialogues at national and international levels, that is bringing together policy makers, civil society and researchers to draw upon evidence and debate key policy questions; training and capacity development for policy makers;

## Methods

This paper analysed the various stages and experiences from seven selected cases where studies conducted by the HPRG were investigated with a specific focus on how the findings from these studies had influenced policy or managerial practice.

The seven cases that represent most of the experiences of HPRG over the years in three States of Nigeria (Enugu, Anambra and Lagos) were selected by the authors to demonstrate the different approaches the HPRG has utilised in seeking to influence policy and/or managerial practice in these states, the important features of these approaches and challenges encountered. These cases were purposively selected from a number of studies conducted by the group because they all show evidence of influencing policy and practice in these States. These cases were not full sample of the group’s recent studies selected but a sample selected to reveal the different types of experience we have. The other studies conducted by the group were excluded because they have not shown any clear evidence of influencing policy and practice. The cases involved a variety of projects, some mainly initiated by researchers and some by policy makers. The authors examined these cases both from the stakeholder/policymaker and researcher perspective.

Fifteen respondents who were purposively selected were interviewed in this study. The 15 respondents were purposively selected because the policy makers and stakeholders were either involved in the various cases or were involved in policy making or were end users of the research findings and in a position to influence policy. The HPRG researchers and other researchers were selected because they were involved in one or more of the studies. Beyond the 15 interviews, the writing team has inside knowledge of these cases and this knowledge is being used in the paper and was tested and extended through the interviews.

### Data collection

In-depth-interviews using an interview guide based on the objectives of the study were used to explore their experiences with reference to the selected cases. Out of the fifteen respondents interviewed, eight were stakeholders/policymakers in various organizations who had different types of engagement with HPRG and six researchers who had been involved in one or more of the cases, and one researcher who is also a policy maker.

Information was collected on stakeholder involvement in generation of research questions, research process (data collection and analysis), generation of results and dissemination of findings. Responses were also collected on the various methods of dissemination of findings employed by the HPRG, and their opinion on which methods that might have worked best for GRIPP. Furthermore, information on factors supporting effective policy engagement and challenges to research utilization in health policy were collected. The responses from the HPRG researchers who were part of the respondents were validated in a feedback meeting after data collection.

### Data analysis

Thirteen of the fifteen interviews were audio recorded and notes were taken. Audio files were transcribed verbatim and subsequently edited. Two interviews were not recorded, but detailed notes were made of the interviews. Conventional content manual analysis (inductive) [27] was used in data analysis. This involved a process of generating of a provisional list of codes/themes that were based on the research questions and study objectives including (stakeholder involvement in generation of research questions, research process, generation of results and dissemination of findings, various methods of dissemination of findings employed by the HPRG, and their opinion on which methods that might have worked best for GRIPP, factors supporting effective policy engagement and challenges to research utilization in health policy); familiarization with the transcripts to identify recurrent/common themes (initial coding); development of the final coding scheme for analysis; application of the coding scheme to the qualitative data; sorting and grouping of coded data to add a more detailed layer of meaning; and exploration of relationships between the themes. All interview transcripts were coded manually by 2 people to reduce inter-coder variability.

## Results

**Fig. 1:**
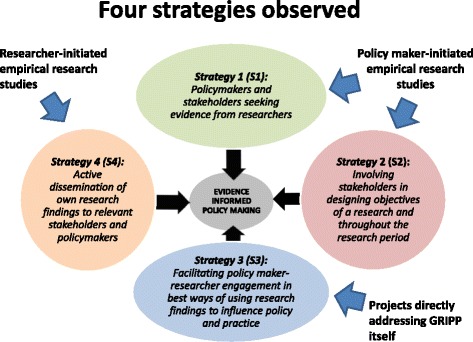
Four Evidence Informed Policy making strategies

The analyses indicated three main ways of engagement between researchers and policy-makers and four more detailed strategies to support evidence informed policy making. This is further presented below and represented in Table 1 and Fig. 1.

**Table 1:** Research-policy engagement groups and strategies

Type of research-policy engagement	Strategies	Project name	Citation	Date of conduct	Geographic location	Main focus
Policy maker-initiated empirical research studies	*Policymakers and stakeholders seeking evidence from researchers (S1)*	Examining appropriate diagnosis and treatment of malaria: availability and use of RDTs and ACTs in public and private health facilities in south east Nigeria	Benjamin SC Uzochukwu, Lausdels O Chiegboka, Chibuike Enwereuzo et al. Examining appropriate diagnosis and treatment of malaria: availability and use of rapid diagnostic tests and artemisinin-based combination therapy in public and private health facilities in south east Nigeria. *BMC Public Health* 2010, 10:486	2009	Enugu	Malaria Diagnosis
		Willingness to pay and benefit-cost analysis of modern contraceptives in Nigeria	Onwujekwe O, Ogbonna C, Ibe O, Uzochukwu B (2013). Willingness to pay and benefit-cost analysis of modern contraceptives in Nigeria: an equity analysis. *International Journal of Obstetrics and Gynaecology*. 122(2): 94–98.	2013	Enugu	Family Planning
	*Involving stakeholders in designing objectives of a research and throughout the research period (S2)*	Promoting universal financial protection: constraints and enabling factors in scaling-up coverage with social health insurance in Nigeria.	Chima Onoka, Obinna Onwujekwe, Benjamin SC Uzochukwu, Nkoli Ezumah. *Health Research Policy and Systems* 06/2013; 11(1):20. DOI: 10.1186/1478-4505-11-20	2012	Enugu and Abuja	National Health Insurance
		*Establishment of Monitoring and Evaluation (M & E) systems for the Anambra Malaria Control Booster Project (MCBP)*	Onwujekwe OE & Uzochukwu BSC. Establishment of Monitoring and Evaluation (M & E) systems for the Anambra Malaria Control Booster Project (MCBP). Project Report	2009–2011	Anambra	Monitoring and Evaluation
*Projects directly addressing GRIPP itself*	*Facilitating policy maker-researcher engagement in best ways of using research findings to influence policy and practice (S3)*	*The PREVIEW (Policy Research EVIdence for Effective Working of the Nigerian health systems) project- Concept and implementation*	Onwujekwe O, Uzochukwu B. 2013. Policy Research Evidence for Effective Working of the Health Systems. Technical Report, Nigerian Academy of Science. www.nas.org	2011/2012	Lagos	Policy maker-Researcher engagement
*Researcher-initiated empirical research studies*	*Active dissemination of own research findings to relevant stakeholders and policymakers (S4)*	CBHI Scheme in Anambra state, Nigeria: an analysis of policy development, implementation and equity effects. (S4)	BSC Uzochukwu, OE Onwujekwe, S Eze, E Nkoli, EN Obikeze and CA Onoka. Community Based Health Insurance Scheme in Anambra state, Nigeria: an analysis of policy development, implementation and equity effects. (www.crehs.lshtm.ac.uk	2006/2007	Anambra	Community-based health insurance policy
		An assessment of policy development and implementation process of District Health System in Enugu state, Nigeria. (S4)	BSC Uzochukwu, OE Onwujekwe, S Eze, E Nkoli, EN Obikeze and CA Onoka An assessment of policy development and implementation process of District Health System in Enugu state, Nigeria (www.crehs.lshtm.ac.uk	2006/2007	Enugu	Decentralization System

The cases selected were categorised into 3 broad types of research-policy engagement: I) Policy maker-initiated empirical research studies; II) Projects directly addressing GRIPP itself; and III) Researcher-initiated empirical research studies (Table 1).

### I. Policy maker-initiated empirical research studies

#### Strategy 1: Policymakers and donors seeking evidence from researchers

In this strategy, stakeholders request evidence on implementation/scaling up. The research is either funded by policy and decision makers or funded by an external agency if the proposal is accepted. Thus the strategy is about being responsive to opportunities created by policy makers calling for evidence. Two cases are used to illustrate this:

1. *“Willingness to pay and benefit-cost analysis of modern contraceptives in Nigeria”*

HPRG was approached by the United Nations Population Fund (UNPF) Nigeria, to conduct a study in order to generate evidence for the national population policy evaluation. The study provided new knowledge on the maximum amount of money that people from different socioeconomic groups and from urban versus rural residential areas was willing to pay for the main contraceptives available in Nigeria and also shed light on factors which explain the WTP decisions in the Nigerian context.

2. *“Examining appropriate diagnosis and treatment of malaria: availability and use of RDTs and ACTs in public and private health facilities in south east Nigeria”.*

Here, the HPRG was approached by the Enugu State ministry of health to assess the availability and use of RDTs and ACTs in public and private health facilities in Enugu.

There is evidence of the usefulness of the results for policy in Enugu State as stated by respondents thus: *“We wanted to know the status of RDTs and ACTs in our State so, we approached HPRG to see if they can come up with something for us as we didn’t have any evidence of what was happening with respect to these malaria control commodities”(Policy maker, Enugu State)*

*“We used the result of availability and use of rapid diagnostic tests and artemisinin-based combination therapy in public and private health facilities study when we were supplying RDTs and ACTs to our health centers………….the study guided us very well and we have RDTs and ACTs all over the facilities now” (Policy maker Enugu State)*

In the two case examples, continuous stakeholder engagement was essential and employed for the effective translation and dissemination of research evidence. Thus beyond the stage of setting the objectives, contact with stakeholders was active and maintained through bi-monthly face-to-face updates on the process.

#### Strategy 2: Involving stakeholders in designing objectives of a research and throughout the research period

In this strategy, stakeholders were involved in the designing of the research and throughout the project period. Two cases are used to illustrate this:

1. *Promoting universal financial protection: constraints and enabling factors in scaling-up coverage with social health insurance in Nigeria.*

The research questions actually arose from the experiences of the Federal Ministry of Health and national health insurance scheme in the adoption or non adoption of the Formal Sector Social Health Insurance Programme (FSSHIP) for their employees. As a result the relevant stake holders and the researchers held meetings and came up with the research objectives. Some members of the Federal ministry of health were also part of the research as data collectors. The results of the study have been widely circulated and it’s now being used by the NHIS to re-design the FSSHIP to ensure the adoption by the States. This was captured by various respondents thus:

*“We are now engaging policy makers at the State level and encouraging them that they do not need to remit their contributions to NHIS in Abuja, but to keep it at the State level to run the State Health Insurance Scheme” (Policy Maker Abuja)*

*“How did NHIS expect us to contribute money and send to them to manage for us. At least now they have seen evidence that we will not do that and they are now asking us to go ahead and establish our own without sending the money to them…. That is the right thing” (policy maker Enugu)*

The active collaboration and participation by the stakeholders facilitated the dissemination and acceptability of the study results of the State adoption of the FSSHIP. This was confirmed by a policy maker:

“*So when the research was about to start it was generally discussed in the stakeholders meetings and some of the questions and the methodology for the research was discussed and we all discussed it and agreed that this is how it’s going to be*…………..*immediately that result was disseminated, we had a change in mind about the FSSHIP………Even other States are following suit” (Policymaker Enugu State)*

2. *“Establishment of Monitoring and Evaluation (M & E) systems for the Anambra Malaria Control Booster Project (MCBP)”.*

Anambra State desired to establish Monitoring and Evaluation (M&E) systems that provide programmatic data in a timely manner for the assessment of the Malaria Booster project. There was an open call to which HPRG responded along with other consultants and eventually won the bid to act as Project Implementation Facilitator (PIF) in Monitoring and Evaluation. HPRG worked closely with the Project Implementation Unit (PIU), M & E Division, State Ministry of Health (SMOH) and other stakeholders, to develop and finalize a harmonized result-based M&E framework for the state MCBP.

With the M&E project, there has been improved M&E framework for both Malaria control activities and other disease programs in the State. The series of briefing and debriefing meetings during and at the end of the project and the participation of key stakeholders in this project facilitated acceptance of the evidence. When asked to give examples of their involvement, one government official said:

*“Myself and some members of the project were involved in the research process and it was effective.”* (*Policymaker, Anambra State)*

The researchers were also of the opinion that the active collaboration of the State ministry of health in both States facilitated the study and made uptake of the results easier. This was captured by two researchers in the following quotes:

*“Positively was the active collaboration with the ministry and other partners in the development of the protocol and implementation of the study as well as good rapport with the partners”……..This enabled them to accept the results (Researcher 2 Enugu)*

“ *In most of those researches, officers of the State Ministry of Health of the two States were involved in every stage of research study starting with generating of research questions to presentation of research findings” (Researcher 3 Enugu)*

There was also a consensus amongst the respondents on the positive influence of involving policy makers in the research process on GRIPP. This was captured by a respondent thus:

“*the robust M&E system the HPRG developed is now being utilized not only by malaria control officers at the local government level but also by the M&E unit of the state ministry of health” (program officer)*

Involving policy and decision makers early on in the research seems to have also enhanced the credibility of the research which is important in its own right. This was captured by some respondents thus:

*“Of course, I mean, the more we involve them , the more credible the results are , the more they can associate, they can relate, some of them say ok, we were part of the research.” (Researcher 2 Enugu)*

*“Yes I was involved in the analyses and generation of study results when I was working with HPRG. I have also done a couple of analyses and generation of study results now in PATHS2. This has affected up-take of study results” (Researcher and stakeholder Enugu)*

### II. Projects directly addressing GRIPP itself

#### Strategy 3: Facilitating policy maker-researcher engagement in best ways of using research findings to influence policy and practice

This strategy is focussed on developing policy maker capacity to use research evidence for policymaking. One case illustrates this:

1. *“Policy Research evidence for effective working of the Nigerian health systems” (PREVIEW) Project.*

The project is a collaborative study between the Nigerian Academy of Science and the Lagos State Ministry of Health. It is directed at stimulating the culture of policy pronouncement, which is based on evidences from research. The project also sought to stimulate the research communities to work in synergy with the policy makers so that health priorities are set following a thorough appraisal and consultative dialogue.

The goal of this project was to enhance institutional capacity among senior and middle level health managers within Federal and State Ministries of Health to use research evidence to influence policy making and improve programmes for effective functioning of the Nigerian Health System using Lagos state as a pilot. Specifically, the objectives of the project were to build capacity of policy makers to utilize evidence for policy formulation and provide a platform to stimulate interaction between the research and scientific community and the targeted policy makers. The project also sought to stimulate the research communities to work in synergy with the policy makers so that health priorities are set following a thorough appraisal and consultative dialogue.

To achieve these objectives, several activities facilitated by the HPRG were employed. A training workshop was held each year for 2 years for middle and senior-level policy-makers in the Lagos State Ministry of health. A training manual (www.nas.org) was developed by HPRG for this purpose.

Two policy retreats including policy dialogues were convened each year for 2 years. At these retreats, researchers, health managers and policy makers interacted and explored i) the opportunity to present the findings of already concluded research and sharing of best practices; ii) identification of priority areas for health research, iii) advocating for the setting up of research data banks and knowledge management units within the Ministries of Health. The retreats were also used as an avenue to encourage research collaboration/networking among the researchers and field managers, as well develop a framework for presentation of the project’s action points and recommendations for improvements and desired best practices to the State Commissioner of Health.

Participants were mainly policy makers, programme managers, local government health supervisors (political appointees), and researchers. In the retreats, policy makers got to know what research evidence existed in their State. Researchers also presented their works and had the opportunity to interact with the policy makers. Discussions at the trainings and retreats revealed that both researchers and policy-makers have a desire to bridge the gap between research, policy and practice. The previous apathy exhibited by both towards each other was identified as a barrier that has militated against attaining significant output and successes needed for improved research uptake for guiding policy direction. The proffered solutions to this was the strengthening of networks in order to jointly source for funding support for implementation research and encouraging partnerships between researchers, decision-makers and other stakeholders. This was succinctly captured by one respondent thus:

*“Well, I think one thing that was critical was the area where we drew up next steps and all the relevant stakeholders were there, including those that do research and policy makers…… to ensure that programmes are evidence-based and guided by research that was identified”(Researcher Lagos State).*

An evaluation at the end of the project showed that many respondents in Lagos State reported improvements in the uptake of research evidence in policy and practices in their work places. These changes include formation of a *Policy maker-Researcher committee* with representatives from tertiary institutions and research institutes and suppository of research evidence in the ministry as captured by some of the respondents:

*“We have formed the Policy maker-Researcher committee domiciled in the State Ministry of Health with representatives from tertiary institutions and research institutes, commissioning research…………..this is a very important milestone” (Researcher Lagos State )*

*“We now have repository of research evidence in the ministry……………researchers now submit there research findings that have to do with Lagos State in the ministry and one can get information about health issues in the State from their”(Policy maker Lagos State)*

Other improvements in the uptake of research evidence in policy and practices in their work places were captured by programme managers and policy makers thus:

*“It was an eye opener and we have used what we learnt to improve practice in the area of childhood immunization, malaria control and non communicable disease control” (Programme manager, Lagos State)*

*“We are now making decisions based on the findings such as with the maternal mortality reduction programmes and policy reviews based on research evidence” (Policy maker Lagos State)*

The participants in this project noted that previously research was not a priority on the list of government’s designed interventions and that researchers/field managers had never collaborated to identify priority areas for research, and were unwilling to share their findings and that governments had never commissioned research nor funded any such activity.

*“Well, I think one thing that was critical was the area where we drew up next steps and all the relevant stakeholders were there, including those that do research and policy makers, we discussed what needed to be done to ensure that research was guiding our programme implementation and how to work out the next steps to ensure that programmes are evidence based and guided by research work that was identified to move the programme forward” -(policymaker Lagos State)*

*“It was a workshop and I was involved. It was like a 2 day retreat, and then we also did one on non communicable diseases. We looked at the stage where Lagos state was at the time concerning having a policy on non communicable diseases. We looked at the policy and tried to bring up some things and we did like a road map on what we should do concerning the non communicable diseases policy and some recommendations were given. One of itwas to revitalize our health research committee and apart from that, all other aspects that we should try and come up with a policy to back our intervention and programme (Policymaker Lagos State)*

Some of the researchers also believed that interacting directly with policy makers would facilitate the use of evidence for policy and improving practice. A Researcher captured it thus:

*“What works best is when you interact directly with the policy makers that will use that evidence, conference papers will not work because they may never read them, so it’s when you interact with them, if you organize something specific for them with them in mind and at the end may be give them policy briefs” (Researcher Lagos State)*

Another policy maker feels that “*retreats and workshops work out better than just writing to inform us of availability of results”*, again buttressing the importance of direct involvement of stakeholders/policymakers.

### III. Researcher-initiated empirical research studies

#### Strategy 4: Active dissemination of research findings to relevant stakeholders and policymakers

In this strategy, the key concepts of knowledge transfer were employed including research reports, peer reviewed papers, conference presentations and policy briefs. The HPRG undertook policy-relevant research and in terms of providing policy advice, HPRG members sought to influence government policy not only indirectly through the publications they produced but also directly through formal means such as participation in government advisory and policy committees, like the National council on Health (highest health policy making body in the country), and contacts with policy makers. Strategies employed to influence policy varied and was influenced by the nature of the policy issue under discussion. In general, this included:

- Production of policy briefs and distribution to policy makers and programme managers.
- Stakeholders’ workshops
- One-on- one discussion of results and advocacy with policy makers and programme managers
- Conference presentations of findings

Examples of the case studies that used this strategy include:

1. *Community Based Health Insurance Scheme in Anambra state, Nigeria: an analysis of policy development, implementation and equity effects (* *www.crehs.lshtm.ac.uk* *)*

The study compared the experiences of two communities that had different levels of success in implementing CBHI in terms of community involvement and support for the scheme and levels of enrolment.

2. *An assessment of policy development and implementation process of District Health System (DHS) in Enugu state, Nigeria. (* *www.crehs.lshtm.ac.uk* *)*

The study investigated the development and implementation of the DHS in Enugu State in order to reveal the underlying factors that affected the implementation of the policy.

The effect of active dissemination of this study included the redesigning of the CBHI programme in Anambra State and adoption of the CBHI in other states of Nigeria. It also led to the review of the district health law of Enugu State.

Most respondents agreed that it is important to disseminate results of research irrespective of the methods used, as it facilitates its use in changing practice. It would seem that an important factor was the active and direct engagement of key stakeholders and policy makers i.e. face to face engagement, supported with written documents as reported by respondents:

*“Yes, by being invited to research findings/results dissemination meeting and capacity building sessions on policy analysis, I got interested in the results…….. was even involved to the extent of convincing some key stakeholders during the dissemination of the research findings/results”(policymaker maker Enugu State)*

*“We usually have dissemination meeting, where the researchers come, we ask questions and at the end of the day, we are able to bring out the issues in the research. That has really helped to influence implementation”-(Policymaker Enugu State)*

When asked to give examples of how the results have influenced policy and practice, policy makers from both States said:

*“The results of the assessment of the Community Based Health Insurance Scheme was very helpful while we were expanding to other communities…..even in the celebrated successful community based health insurance in Igbo-Ukwu we used the HPRG results to make it work better”(Policy maker Anambra State)*

*“We have started reviewing the district health law to capture some of your recommendations” (policy maker, Enugu State)*

### Factors supporting effective policy engagement by the HPRG and getting research into policy and practice

Several factors were critical in supporting effective policy engagement by the HPRG and getting research into policy and practice. These included five aspects:

1. *Willingness of Policy makers to use research findings even if they go against their expectations or against current policy*. This fact was buttressed in Enugu State and was captured by a policy maker thus:

*“there is willingness of the users to use the research findings and that is why it’s important to enter into a form of commitment agreement that whatever the findings will be that you will make use of them.” (Policy Maker Enugu)*

2. *Credibility of both the research findings and the HPRG researchers can and do influence GRIPP.* Examples of statements in this category are:

*“Through a long standing cordial relationship with the HPRG, we have found them to be credible and hence feel comfortable using their research findings to impact policy and practice” (Policy Maker Enugu State)*

*“We appreciate your works because you publish in international journals of high impact value” (Policy maker, Enugu State)*

3. *Relationship and Trust*.

Close and long relationship between the HPRG researchers and policy makers particularly in Anambra and Enugu States facilitated GRIPP. One respondent referred to this contribution thus:

*I got to know HPRG from their presentations in conferences and their contributions to policy issues in Nigeria especially in the national council on health……………… (policy maker Lagos)*

4. *HPRG networks.*

Linkages to policy makers like the Enugu Forum (a forum where policy strategic issues are discussed on bi-monthly bases) helped promote policy influences. Also Linkages with international consortia in terms of south-south and south-North collaboration helped in generating evidence. As noted by one of the HPRG researchers:

*“I think because HPRG belong to several reputable international consortia, they have good opportunities of conducting country researches that are relevant to policy in Nigeria”*

5. *International agendas drive domestic policy making.*

This was clearly the case with the UNFPA study in which international interest was evident. International agendas driving domestic policy making has implication for researchers as they try to link research to international agendas. A policy maker noted that internal interests on an issue facilitate getting evidence into practice as captured thus:

*“Yes, there was this international agenda of the MDGs, you know the Free MCH services actually is targeting the women and children addressing MDG 3 & 4…… and once that policy was formulated because it has an international component, the government was interested in it and that was why it sailed faster than any other policy we have ever formulated in this state” (Policymaker Enugu State)*

*“If those studies are addressing current global or national conversations, something that is topical, i.e. everyone is talking about it, if for example you generate evidence on universal coverage, or polio eradication, or maternal and child health, you know, topical or a state or the national level is trying to develop a policy or strategic framework, and it fits into it…….” (HPRG Researcher)*

### Challenges to research utilization in health policy identified by HPRG in the course of their work

There were also challenges to research utilization in health policy, which HPRG identified in the course of their work and these were also highlighted by the respondents during the interview. These challenges included:

1. *Capacity to use HPSR*

This is in terms of decision-makers demand for and uptake of HPSR; their research uptake skills and its practical implications for evidence-based policy making. It was noted that some decision makers lacked research uptake skills and in the word of a researcher. The quote below illustrates this:

*“One of the problems of demanding evidence and uptake of results is that the policy makers hardly appreciate the importance of this and they simple lack the skills to do so. They don’t even know whom to turn to if they need information to underpin a policy decision …..hence there is need to train them on this” (Researcher Lagos State)*

This was further echoed by a policy maker: *“if we are trained on a regular basis on the need to recognise when to ask for the results and how to interpret them, things will go on smoothly” (Policy maker Lagos State)*

2. *Communication gap between researchers, donors and policymakers.*

Communication gap between researchers, donors and policy makers seem to present a challenge to research utilization. In the words of one of the respondents:

*“There is also a communication problem between the donors and the researchers because the donors want a particular thing to be researched which might not be the crux of the problem” (Researcher Lagos State)*

3. *Managing the political process of GRIPP.*

According to the respondents, if work is commissioned, then results need to be made available in a timely way. But if work is not commissioned it is pure luck if a study being done is relevant to the topic of focus and completed on time to influence a particular debate. Respondents captured this thus:

*“I think researchers themselves need to be politically minded to know when to produce evidence and how to get it across to the users, given the various interests of policy makers and their disposition”. (HPRG Researcher).*

*“A researcher must have knowledge of the most pressing political and policy questions that they would need to make their research more relevant and be connected to the politicians and policy makers in one way or the other” ( HPRG Researcher ).*

4. *Lack of willingness of some policy makers to use research.*

This is greatly influenced by the political context within country and not always easy to change. This is captured by a respondent

*“I think the biggest problem is the resistance to change. This is because, we will do some surveys and you try to tell people that this is what we found in this survey and people will say no, no, this is how we have been doing it.” (Researcher 1 Enugu)*

5. *Limited research funding and resistance to change*

Limited research funding and resistance to change were some of the constraining factors offered by respondents which would affect GRIPP. This was captured by several quotes from the respondents.

*“..from the local point of view, governments of states are not always interested in research. In most cases little or no budgets are made for research. Even where there are fiscal releases they are hardly used for research”- (Researcher/stakeholder Enugu State)*

*“Well when you look at the cost, at times the state will say these people who are proposing this are they going to fund it? Once they start like that, putting cost before effect, you know you are not going to get anywhere.”-(Policymaker, Enugu State)*

## Discussion

Four key strategies to getting research into policy and practice have been discussed using HPRG experiences namely: policymakers and donors seeking evidence from researchers where stakeholders request evidence on implementation/scaling up; involving stakeholders in designing objectives of a research and throughout the research period. Others included facilitating policy maker-researcher engagement in best ways of using research findings to influence policy and practice which focussed on developing policy maker capacity to use research evidence for policymaking and active dissemination of research findings to relevant stakeholders and policymakers.

The experiences of the HPRG have shown that the involvement of policy and decision makers early on in the research-identifying and setting research priorities process and then also through data collection appeared to have enhanced evidence to policy and practice. This is consistent with findings from other developing and developed countries [28, 29]. It has also been noted that policy makers usually are well aware of the importance of evidence based policy and will be prepared to consider evidence in policy making, but would like to be involved in the process of evidence generation [30]. This also calls into play the willingness of policy and decision makers to understand and interpret evidence required to make policy as stated by some of the respondents.

Of all the HPRG GRIPP strategies, collaboration of researchers with policy makers is key and it has been noted as being helpful in translating research into policy in this study, but this may only be possible when the policy makers are made a part of the research process from the onset as in strategy 2 rather than giving them research findings they were not involved in.

It was discovered that in terms of potential to influence domestic decision-making there was differences between externally funded work and domestically commissioned work as both results were used to improve practice. There was a perception in this study that donor partners can often promote their ‘preferred’ studies which received their financial or technical support.

The method of presentation of research findings by researchers to policy makers, also influenced the research uptake as have been noted in this study. A majority of the respondents (researchers and policymakers) were of the opinion that this could be greatly enhanced by the involvement of the end users of the research findings early in the research process and at the time of dissemination. They also opined that findings be presented in simple and easy-to-understand language. These solutions have also been suggested by other studies, both in Nigeria and in other contexts [28, 31].

When it comes to the dissemination stage of a research, the methods chosen should be most suited to the target audience. While some of our respondents felt that the dissemination method may not be a factor, a majority felt that active engagement of end users and policy makers during knowledge transfer would greatly impact its translation into policy and practice. Direct engagement i.e. face to face engagement, supported with written documents, was the most popular preferred method of dissemination amongst our respondents. It is important to note that the information should be summarized and packaged in ways that policy-makers and civil society representatives can use, while hoping that policy-makers have sufficient capacity to access and apply research findings. This has also been stressed in various studies in Indonesia, Tanzania and Nepal [28].

Although the HPRG produces policy briefs for policy makers from its researches, it also publishes in international peer-reviewed journals of high impact value and this has been appreciated by the policy makers and has enhanced GRIPP. Some authors have noted that an additional mechanism to help maintain scientific credibility and demonstrate strong technical quality is through publishing findings in peer-reviewed journals [32].

HPRG facilitated the knowledge brokering process between policy makers and researchers in Lagos State so that together they can produce research informed policy options. This encouraged policy makers and program managers to be more responsive to research findings, as exemplified by the formation of a researcher-policymaker committee and the establishment of a suppository of research in the State ministry of health. It also stimulated researchers to be aware that they need to conduct policy-relevant research and produce the evidence in a format that is reader-friendly to the policy makers. Thus bringing together decision makers and researchers who can engage with one another and use research evidence in policy formulation is, one of the important strategies for seeing that the findings are applied. This fact has also been noted elsewhere [7, 33, 34].

Several factors were critical in supporting effective policy engagement by the HPRG and getting research into policy and practice. These included: willingness of policy makers to use research findings even if they go against their expectations or against current policy, credibility of the research findings, HPRG researchers’ relationship and trust and HPRG networks.

Linkages to policy makers like the Enugu Forum (a forum where policy strategic issues are discussed on bi-monthly bases) helped promote policy influences. This forum discusses articles in academic journals, and peer discussion on contemporary health issues in Enugu State and environment. It has membership from policy makers, CSOs, media practitioners, politicians, economists, business moguls, researchers and academia and offered the HPRG members the opportunity to share their research findings.

Also Linkages with international consortia helped in generating evidence. The HPRG has been a member of several international consortia over the years, like Consortium for research in equitable health systems (CREHS), Consortium for health policy and systems analysis in Africa (CHEPSAA), Resilient and Responsive Health Systems (RESYST) and through their relationships and conducting research together has facilitated the generation of relevant results for policy making.

International agendas driving domestic policy making has implication for researchers as they try to link research to international agendas. It is important to manage the political process of GRIPP. From our experience it was discovered that if work is commissioned as in strategy 2, then the results need to be made available in a timely way. But if work is not commissioned as in strategy 4, it is pure luck if a study being done is relevant to the issues of focus and completed on time to influence a particular debate. The challenge therefore is usually for researchers to make themselves available to participate in policy processes on top of all other work and to draw in older research to current debates. The issue then is more about being able to relate past research findings to current debates. Also of importance is the powerful influence of politicians and role of other stakeholders as this can constrain GRIPP.

It is important to note that one of the enabling factors in getting the HPRG research results into policy and practice is the close and long relationship between the HPRG researchers and policy makers particularly in Anambra and Enugu States. Personal links between HPRG members and policy makers has played critical role in fostering trust and influence. Thus getting research into practice is greatly influenced by the credibility of the research findings and the researchers, as well as the relationship and trust between the researchers and policy makers. Some policymakers in this study stated that through a long standing cordial relationship with the HPRG, they have found them to be credible and hence feel comfortable using their research findings to impact policy and practice. Interpersonal relationship has been stressed as a good way of strengthening the relationship between the researcher, policy makers and the practitioners and thus, bridging the gap among them [33].

There were also challenges to research utilization in health policy, which HPRG identified in the course of their work and these were also highlighted by the respondents during the interview. These challenges included capacity to use HPSR in terms of decision-makers demand for and uptake of HPSR; their research uptake skills and its practical implications for evidence-based policy making. Training policy makers and programme managers on these will enhance research uptake. There were also communication gap between researchers, donors and policymakers which presented a challenge to research utilization. This can be improved by appropriate information education and communication strategies.

Furthermore, managing the political process of GRIPP was an issue. According to the respondents, if work is commissioned, then results need to be made available in a timely way. But if work is not commissioned it is pure luck if a study being done is relevant to the topic of focus and completed on time to influence a particular debate. The challenge therefore is for researchers to make themselves available to participate in policy processes on top of all other work and to draw in previous research to current debates. The issue then is more about being able to relate past research findings to current debates. And as stated by a respondent, “*researchers themselves need to be politically minded to know when to produce evidence and how to get it across to the users, given the various interests of policy makers and their disposition”.*

Lack of willingness of some policy makers to use research was also found to be a challenge and this is greatly influenced by the political context within country and not always easy to change. Finally, limited research funding and resistance to change were some of the constraining factors offered by respondents which would affect GRIPP underscoring the need to improve funding for research in the country.

## Conclusions

With the right level of interaction between researchers and decision-makers, the translation of research findings into actionable policy and programmatic guidance is an achievable goal. Country HPSR groups can influence domestic policy makers if appropriate strategies are employed. This paper has tried to bring together in the Nigerian context a preview of positive features and challenges in the process of getting research findings into policy and practice through the investigation of seven cases and how the involved policy makers and researchers of an institutional-based HPSR group experienced these cases. Our experience suggests that four strategies converge to create pathways through which research can get into policy and practice. Depending on the policy under consideration, any of the strategies or a combination of them can be employed. Much of the work undertaken by the HPRG was driven by requests from government or donors and the primary outputs were research reports, journal publications, policy briefs and verbal briefings through feedback workshops and one-on one briefing**.**

The integration of research findings into policy and communicating research findings to Nigerian policymakers is necessary if improved policy decisions are to be adopted, especially within the context of universal health coverage. It requires a deep understanding of how to interact with policymakers, what information they require, and in what form and with whom to establish interactions

It is necessary to educate decision makers and practitioners about the relevance of evidence produced, as exemplified in some of the strategies. It is also necessary to develop context specific sub-strategies and activities that can explain how the findings can be utilized in practice. Interpersonal relationship and trust, and good networks are helpful ways of strengthening the relationship between researchers, policy makers and practitioners.

Respondents uniformly agreed that research findings need to be timely, i.e. to be made available when they are needed to influence policy and practice. In addition a majority also felt that if the research question was a topical issue in a given context, that it would also positively influence GRIPP. Some of the challenges to research utilization in health policy found to be important was the capacity of policy makers to demand for and uptake research, communication gap between researchers, donors and policymakers, managing the political process of GRIPP, lack of willingness of some policy makers to use research and limited research funding and resistance to change.

It s also important to consider the factors that enable and constraint getting research into practice and policy as noted in this study in bridging the gap between researchers and policy makers.

### Limitations of the study

Our sample of policy makers and researchers was not intended to be representative of the whole country, but rather those who have a practical range of views from their experience with the HPRG. The GRIPP-strategies derived from the seven cases that together represented a variation of stakeholders, subject, interventions and activities can be compared with additional cases in other contexts to verify strategies and their impact. The roles of the media and donors in bridging the gap between researchers and policy makers were not evaluated in this study. This will form a basis for a further study.

## Abbreviations

ACTs: Atemisinin-combined therapy
CBHI: Community Based Health Insurance
CHEPSAA: Consortium for Health Policy & Systems Analysis in Africa
DHS: District Health System
FSSHIP: Formal Sector Social Health Insurance Programme
GRIPP: Getting research into policy and practice
HPRG: Health Policy Research Group
HPSR: Health Policy and Systems Research
IDIs: In-depth-interviews
LMIC: Low and middle-income countries
M&E: Monitoring and Evaluation
MCBP: Malaria Control Booster Project
MCH: Maternal and Child Health
MDGs: Millennium development goals
NDHS: Nigeria Demographic and Health Survey
NHIS: National Health Insurance Scheme
NIMR: Nigerian Medical Research
PATHS2: Partnership For The Transformation Of Health Systems
PIF: Project Implementation Facilitator
PIU: Project Implementation Unit
PREVIEW: Policy Research evidence for effective working of the Nigerian health systems
RDTs: Rapid Diagnostic Tests
RESYST: Responsive and Resilient Health Systems
SMOH: State Ministry of Health
TDR: Tropical Disease Research
UNFPA: United Nations Fund for Population Activities
